# Effect of Different Seasons and Development Stages on the Chemical Composition and Bioactive Potential of Cardoon

**DOI:** 10.3390/foods13162536

**Published:** 2024-08-14

**Authors:** Filipa Mandim, Márcio Carocho, Spyridon A. Petropoulos, Celestino Santos-Buelga, Lillian Barros

**Affiliations:** 1 Centro de Investigação de Montanha (CIMO), Instituto Politécnico de Bragança, Campus de Santa Apolónia, 5300-253 Bragança, Portugal; filipamandim@ipb.pt (F.M.); lillian@ipb.pt (L.B.); 2 Laboratório Associado para a Sustentabilidade e Tecnologia em Regiões de Montanha (SusTEC), Instituto Politécnico de Bragança, Campus de Santa Apolónia, 5300-253 Bragança, Portugal; 3 Grupo de Investigación em Polifenoles (GIP-USAL), Facultad de Farmacia, Universidad de Salamanca, Campus Miguel de Unamuno, 37007 Salamanca, Spain; csb@usal.es; 4 Laboratory of Vegetable Production, Department of Agriculture, Crop Production and Rural Environment, University of Thessaly, Fytokou Street, 38446 Volos, Greece; spetropoulos@uth.gr

**Keywords:** phenological stage, plant adaptation, eco-evolution, meta-analysis, chemometrics, *Cynara cardunculus* L.

## Abstract

*Cynara cardunculus* L. (cardoon) is a wild species of the Mediterranean basin and is highly appreciated due to its rich nutritional value and versatile industrial applications. It is widely known that environmental conditions, such as air temperature, humidity, and solar radiation, among others, play a crucial role in plant phenological variations and the chemical composition and bioactive properties of different plant tissues of cardoon. This study applied several statistical methods to uncover the variations in biomolecules of different cardoon tissues collected in Greece over the growth cycle. The influence of the different seasons on the species is evident, resulting in a clear discrimination between the samples harvested throughout the growth cycle. In addition, the observed fluctuations in chemical composition are consistent with each vegetable tissue’s functions and the plant’s different physiological processes. This work allows for a better understanding and knowledge of the species, encouraging more profitable and sustainable use of all the plant parts.

## 1. Introduction

The sustainable and proper use of planetary resources is one of the major global concerns nowadays [1], and therefore the study of vegetable species and the implementation of sustainable strategies for proper crop management is extremely necessary. The identification of relevant plans of action that can be adopted at local, national, and international levels with simultaneous economic, environmental, and social impact is essential to achieving sustainable goals and ensuring the needs of current and future generations [2,3].

Biotic and abiotic factors influence the physiological and morphological aspects of vegetable species, and thus, the plant’s response to these external factors is complex and exhibits distinct characteristics inherent to each vegetable species [4]. These phenomena have a huge impact on primary and secondary metabolism, strongly influencing almost all phenotypical parameters. They also interfere with the biosynthesis and accumulation of different classes of compounds (e.g., sugars, carbohydrates, fatty acids, phenolic compounds, terpenes, and alkaloids, among others) [5,6].

Despite the large consumption of plant species around the world, their management in the agri-food supply chain is a multifaceted process [2]. The difficulties are commonly related to their short shelf-life and to the fact that large amounts of biomass generated during cultivation are not being properly handled. Based on this, knowledge and complete characterization of vegetable species are necessary to help achieve higher profitability and increase the added value of crops. This knowledge also allows the reduction of discarded vegetable material [7,8,9]. In an attempt to better understand the conditions that influence the chemical composition and, consequently, the biological properties and applications of different vegetable species, several studies have been developed, although their focus is mostly on the analysis of tissues in one specific phenological stage (most commonly at the harvesting stage of marketable products), which provides the fingerprint of the different biomolecules at that particular time [10]. The application of various statistical tools offers an innovative approach to multidirectional data analysis, allowing in-depth visualization and interpretation, identifying patterns of variation in the variables under study, and identifying specific characteristics associated with the data under analysis.

*Cynara cardunculus* L. (syn. cardoon) is widely distributed in the surrounding countries of the Mediterranean basin, is used in folk medicine and several traditional recipes, and is appreciated for its rich phytochemical and nutritional composition [11]. The cardoon plant organs are known to be a rich source of biomolecules such as caffeoylquinic and dicaffeoylquinic acid derivatives, or luteolin and apigenin glycosides, among others [12,13,14]. This crop also has applications in different industrial sectors, such as paper pulp, biodiesel, biofuel, and biomass production [11,15,16]. Its chemical composition and bioactive properties have been widely described, and several studies have emphasized the meaningful influence that edaphoclimatic conditions have on those parameters [17,18,19]. However, the combined application of statistical methodologies that provide a multidimensional approach to the data and, consequently, to the biochemical variations in the different plant tissues of cardoon throughout the growth cycle is a study that can provide a wide variety of information that has not yet been developed.

The present study intends to understand how the chemical composition and bioactivities of *C. cardunculus* plant parts vary throughout a complete growth cycle. For this purpose, several statistical methodologies were used to verify which variables are affected the most by the different seasons and development stages, therefore allowing in-depth visualization and interpretation of biochemical variations.

## 2. Materials and Methods

### 2.1. Plant Material

**Figure 1:**
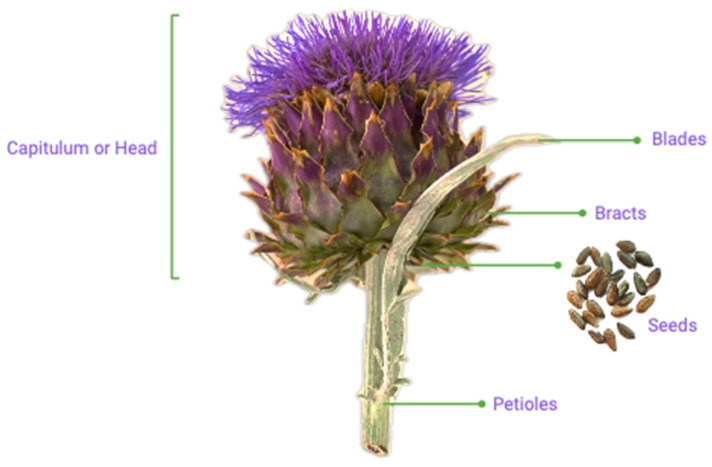
Representation of the studied cardoon (*Cynara cardunculus* L.) plant tissues.

*Cynara cardunculus* var. *altilis* DC. cv. Bianco Avorio (Fratelli Ingegnoli Spa, Milano, Italy) tissues were collected at the experimental field of the University of Thessaly, in Velestino, Greece (22.756 E, 39.396 N) between September 2017 and August 2018. The studied parts were the heads, blades, bracts, petioles, and seeds (Figure 1). The harvesting months of the different plant tissues studied, as well as the principal growth stages (PGS), according to the Biologische Bundesanstalt, Bundessortenamt, Chemische Industrie (BBCH), scale are summarized in Table 1. The biotic conditions throughout the growing season and the collection methodology have been previously described by Mandim et al. [20], allowing for a complete analysis over 12 months for this work.

### 2.2. Studied Variables

The different plant tissues of cardoon harvested throughout its development cycle have been extensively characterized for their chemical composition and bioactive properties in previous studies by our team. The lipidic fraction and the composition of fatty acids, free sugars, tocopherols, and organic acids were previously analyzed using the appropriate chromatographic methodologies for each class of compounds [19,20,21,22,23]. The hydroethanolic extract of each of the samples was obtained and characterized for its phenolic composition and bioactive properties. The anti-inflammatory, antiproliferative, antioxidant, and antimicrobial capacities have also been studied following the respective protocols [18,22,24,25,26]. The articles from which the data for this manuscript were extracted included several biomolecules from primary and secondary metabolism, namely individual fatty acids, tocopherol isoforms, individual phenolic compounds, organic acids, and soluble sugars. Furthermore, these studies also evaluated several bioactivity parameters, namely antioxidant, anti-inflammatory, and antiproliferative activities, as well as hepatoxicity assays. The antioxidant assays included the inhibition of thiobarbituric acid reactive species (TBARS) and oxidative hemolysis inhibition (OxHLIA). The antitumor effects were tested against HepG2, HeLa, NCI-H460, and MCF-7 cell lines. The cytotoxicity analysis included the use of PLP2 cell assays, while the anti-inflammatory activity relied on the RAW 264.7 cell line assay [18,19,20,21,22,23,24,25,26]. All the cell lines used were commercially acquired from authorized cell line resources namely the German Collection of Microorganisms and Cell Cultures (DSMZ) and the European Collection of Authenticated Cell Cultures (ECCAC). To maintain high scientific standards, all procedures will be performed according to the best practices observed in the Guidance on Good Cell Culture Practice (GCCP). For better clarity and transversality of the statistical analysis of the results obtained from different vegetable tissues, only the parameters that were detected for all the tissues were considered.

### 2.3. Statistical Analysis

Several statistical tools were used to classify, group, and discriminate between the different tissues throughout the 12 months of analysis. Each tissue of the cardoon separately underwent the same statistical analysis. Initially, a classification was performed with a linear discriminant analysis to understand how the different months were related to each other based on the variation of the different data. Then, a principal component analysis was carried out to understand if clustering by biomolecule family was possible. Following the clustering analysis, a dendrogram classification was performed to understand how each parameter related to each other. A heatmap analysis was performed, showing how each specific parameter in each tissue varied over time. Finally, a Pearson’s correlation analysis was also performed to evaluate the correlation between the chemical composition and the studied functional properties. All statistical analyses were performed with SPSS Statistics software (IBM SPSS Statistics for Mac OS, Version 26.0; IBM Corp., Armonk, NY, USA) and Prism 10 software for MacOS, version 10.0.1 (GraphPad, San Diego, CA, USA). The comparison of the averages was previously performed by a one-way analysis of variance (ANOVA), followed by Tukey’s HSD test (α = 0.05). A student’s *t*-test was applied when only two samples were compared [18,19,20,21,22,23,24,25,26].

#### 2.3.1. Linear Discriminant Analysis

A linear discriminant analysis (LDA) was applied to discriminate between the harvest stages of each of the cardoon tissues. For the LDA analysis, Wilk’s λ test was applied using the stepwise method, with an F-value of 3.84 for entering and 2.71 for removal employing the leave-one-out cross-validation procedure. The matrices displayed show the within-group correlation [27].

#### 2.3.2. Principal Components Analysis

The principal components analysis (PCA) was used to evaluate the contribution of the studied variables (chemical composition and bioactive activities) to the total diversity, clustering them, when possible, in families. The number of dimensions for each analysis was assessed by the respective eigenvalues, obtained from a scree plot. The chosen factor analysis was the principal component with a direct oblimin rotation using a Kaiser normalization and sorting of components by size [28].

#### 2.3.3. Cluster Analysis

For the clustering analysis, two clustering methods were used. The first is a hierarchical cluster analysis (HCA), which outputs a dendrogram that shows the proximity of each parameter to each other. The chosen clustering method was Ward’s method, which allows for equal-sized clusters using a Z score standardization to overcome the different magnitudes of the data. For further confirmation, a two-step cluster analysis (2SC) was used to tentatively confirm if the chosen number of clusters was correct for the HCA. In this method, the clusters were defined automatically using Schwarz’s Bayesian criterion and a log-likelihood for the distance of cluster memberships. The log-likelihood was chosen because not all data were normally distributed, which is a prerequisite for Euclidean distance membership. After confirming that the number of clusters in 2SC was the same as the ones in the HCA and that the quality of the clusters was at least “fair” (options are good, fair, and poor), the number of clusters was interpreted [29,30].

#### 2.3.4. Heatmaps

Heatmaps are two-dimensional charts that separate the magnitude of data according to a color pattern. Once again, due to the different magnitude of the data present in each parameter, the heatmaps used in this manuscript allowed for a visualization of the different parameters of each tissue over time, granting an understanding of their increase or decrease throughout the growth cycle. Due to the difference in the magnitude of the data, a normalization step was performed before the generation of the heatmaps. In the heatmap figures, higher values are represented with darker tones, while lower ones are shown with lighter ones [31].

#### 2.3.5. Pearson Correlation

A Pearson correlation coefficient between the functional properties of the studied samples and the chemical compounds identified, including the sums of the different classes, was performed with a 95% confidence level.

## 3. Results and Discussion

### 3.1. Linear Discriminant Analysis

Linear discriminant analysis (LDA) aims at finding a linear projection that maximizes the ratio of the between-class variance. In this particular case, the LDA was used to verify the differences associated with seasonal variations of several parameters of 5 cardoon tissues, namely heads, seeds, petioles, bracts, and blades.

**Figure 2:**
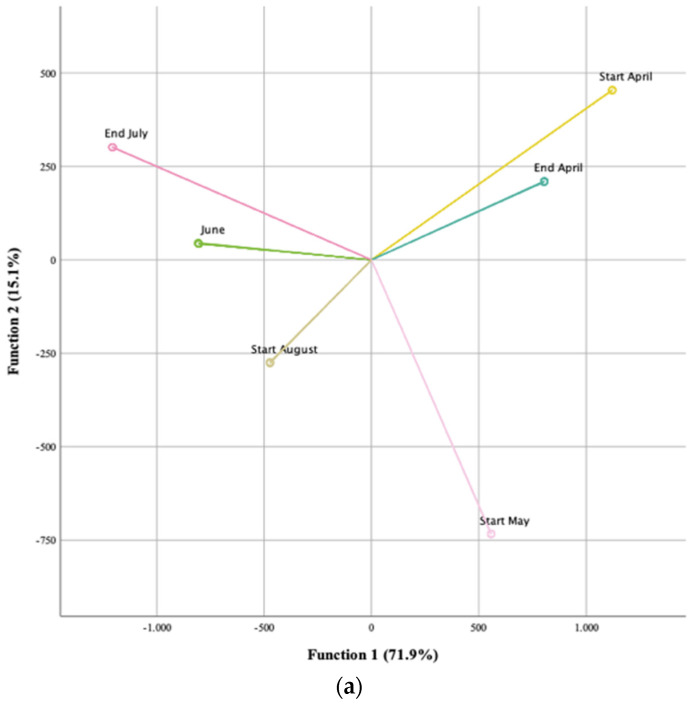

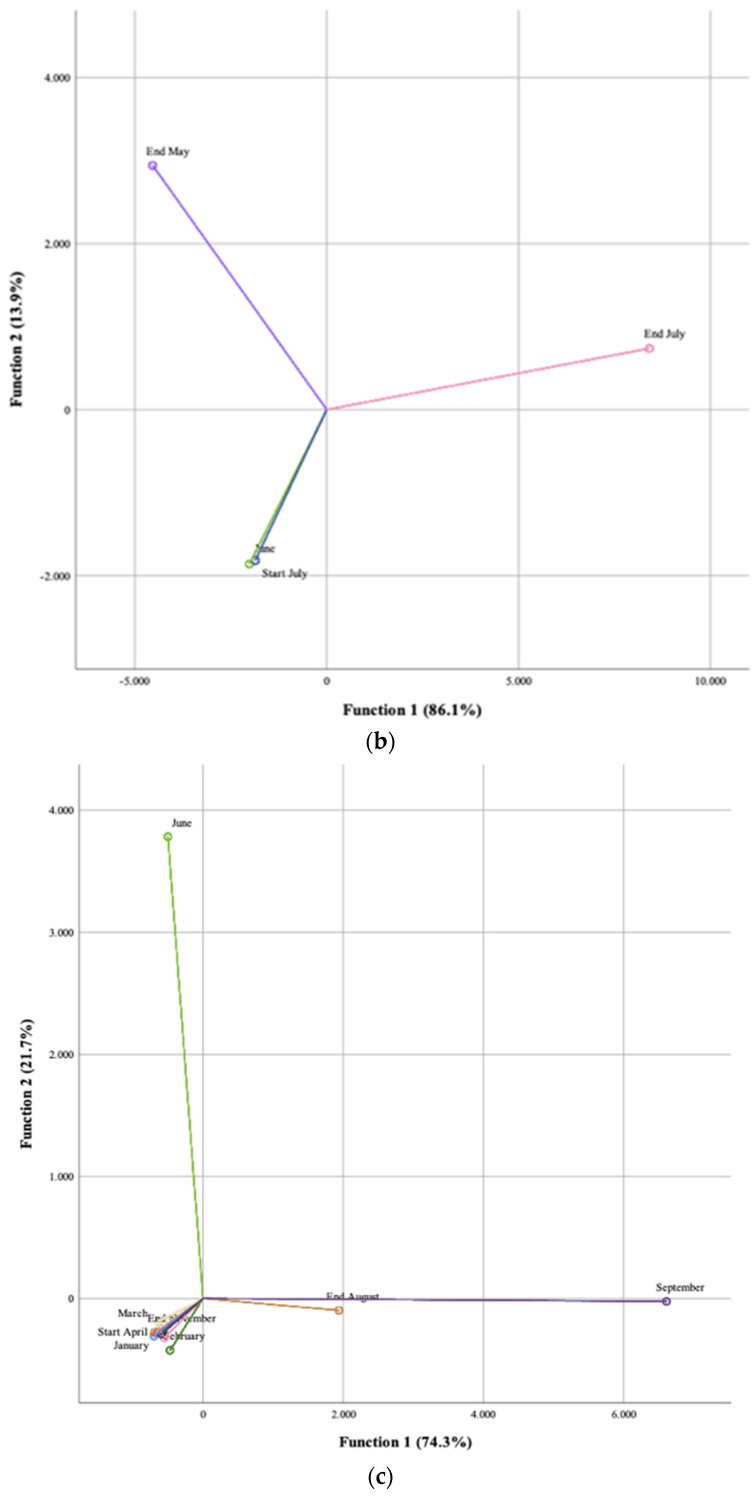

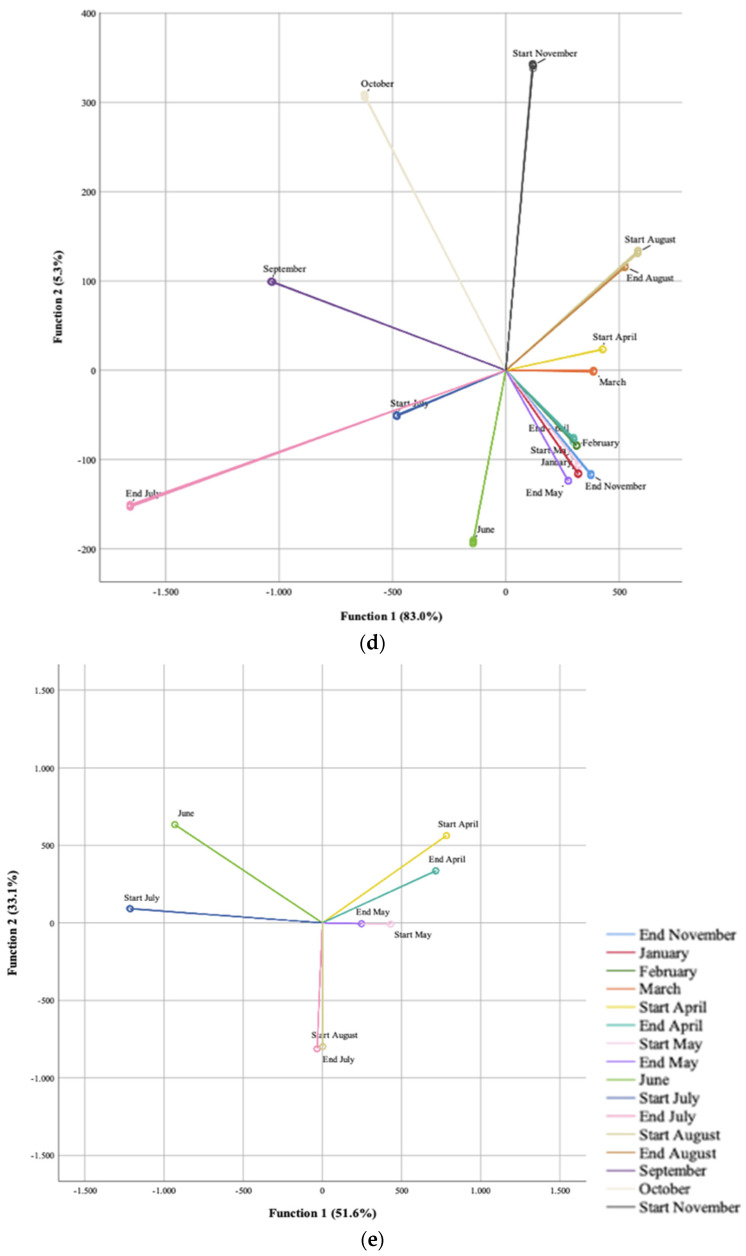
Linear discriminant analysis (LDA) of the *Cynara cardunculus* L. vegetable tissues: heads (**a**), seeds (**b**), petioles (**c**), blades (**d**), and bracts (**e**). Information regarding the harvesting months of the plant tissues studied is summarized in Table 1.

For cardoon head samples, 6 different harvesting times were analyzed, from the beginning of April to the beginning of August, due to the heads of cardoon only being present during this time period of the year. The model defined five functions that accounted for 100% of the variability, the first two being responsible for 87% (function 1–71.9%, function 2–15.1%) (Figure 2a). Out of a total of 28 parameters, 18 were included in the model as being the best predictors and having the highest discriminating ability. The five most discriminant parameters were C16:1 (palmitoleic acid), C:6 (caproic acid), C:17 (stearic acid), total flavonoids, and C22:1 (erucic acid). Interestingly, four of the five most discriminating parameters were individual fatty acids. For both function 1 and function 2, the variable with the highest correlation was linolenic acid. The plot of the cardoon heads presented in Figure 2a shows that function 1 clusters the first two harvesting months of April and May with the three final months of June, July, and August. This clustering accounted for almost 72% of the total variation. To a lower degree, function 2 showed that May presented a bigger difference from the rest of the months, placing this month near the bottom right corner of the plot. Overall, the heads of cardoon are discriminated by the lipidic fraction and tend to vary in two distinct stages, namely the first three months when they are formed and harvesting of marketable product takes place and the final three months before senescence and dying out.

Cardoon seeds were analyzed from May to the end of July, due to the short time period they were present on the plant. The plot of the LDA for the seeds is shown in Figure 2b. The model defined two functions responsible for 100% of the variability (function 1–86.1%, function 2–13.9%). Of the 32 parameters analyzed, only 9 were included in the statistical analysis. The parameters that exhibited the highest discriminant power, meaning they are the best predictors for the model, were C18:3n3 (linolenic acid), α-tocopherol, total organic acids, C11:0 (undecanoic acid), and C20:0 archidic acid. The tocopherol content and the C20:0 fatty acid were the variables with the highest correlation within function 1. On the other hand, in function 2, it was the lipidic content and linolenic acid that exhibited the highest correlation. By analyzing Figure 2b, a clear discrimination between the different harvesting dates is visible. Function 1 discriminates seeds harvested at the end of July from the rest of the sampling dates, while function 2 discriminates samples harvested between the end of May and the beginning of July and June. Cardoon seeds collected at the end of July exhibited a higher difference from the rest of the samples, which was mostly associated with the tocopherol and C20:0 fatty acid contents. The harvest between June and July does not exhibit pronounced differences since they are practically overlapping. This shows that between June and the beginning of July, the changes in the analyzed parameters do not vary considerably, although before and after this period, the variation is quite pronounced.

Petioles and blades are present during the whole year, so their analysis included samples collected throughout the growth cycle. Fifteen functions were defined for petioles (Figure 2c), the first two being responsible for 96% of the variability (function 1–74.3%, function 2–21.7%), while the other 13 showed residual variance. Of the 42 parameters tested, only 24 were included by the statistical method, being α-tocopherol, C20:5n3 (eicosapentaetanoic acid), C12:0 (lauric acid), C18:1n9 (oleic acid), and total phenolic compounds, those with the highest prediction potential. The content of α-tocopherol was the variable that exhibited the highest correlation within function 1, and eicosapentaetanoic acid within function 2. In the plot of petioles (Figure 2c), it is clear that samples collected in June and September are discriminated by both functions from the rest of the harvesting months. The differences observed in samples harvested at the end of August and September, discriminated by function 1, are justified by the α-tocopherol content (higher at that time), while the petioles harvested in June are discriminated by function 2 due to C20:5n3 content.

The difference between these two sampling points, representing the starting and ending dates of the analysis, and the other months is quite remarkable, showing drastic shifts at the beginning and the end of the season. The remaining harvesting dates are positioned very close to each other, showing very low variation in their composition.

Considering the cardoon blades (Figure 2d), once again 15 functions were defined, with the first two being responsible for 88.3% of the variability (function 1–83% and function 2–5.3%). From a total of 37 parameters, the model included 30 with higher discriminating capacity. The five that better predicted this model were α-tocopherol, total flavonoids, total soluble sugars, total organic acids, and C23:0 (tricosanoic acid). Interestingly, the blades of cardoon, unlike the previous tissues studied, were not discriminated by individual fatty acids but rather by three different groups of compounds, namely polyphenols (flavonoids), soluble sugars, and tocopherols. Function 1 exhibited a higher correlation with total phenolic acids and total flavonoids, while function 2 better correlated with total flavonoids. Considering the plot for the cardoon blades, the different months are spread out over the two dimensions. Still, function 1 completely discriminates the end of July from the other months, while function 2 discriminates August, September, October, and November, which could be due to the gradually decreasing temperatures. These discriminations are highly reliant on shifting polyphenols (gradually increasing), as the different groups of these secondary metabolites are the best predictors of the model. The other analyzed months are mostly grouped in clusters close to each other.

Finally, the LDA of cardoon bracts defined seven functions (Figure 2e), with function 1 and function 2 being responsible for 84.7% of the observed variability (function 1–51.6% and function 2–33.1%). Only 17 of 39 parameters were included in the stepwise model, with C15:1 (pentadecanoic acid), total phenolics, C18:0 (stearic acid), oxalic acid, and total lipids being the 5 best predictors. The total lipidic content and total phenolic compounds were the variables with the highest correlation within function 1. For function 2, the parameters better correlated were total phenolic compounds. From the interpretation of Figure 2e, function 1 clusters the months into two groups of consecutive months. June and July are clustered on the left, while April and May are clustered on the right side of the plot. August and July are clustered between the two horizontal clusters but are better discriminated by function 2. This difference can also be explained by the higher content of phenolic compounds in the samples collected in July [22].

Overall, the different analyzed tissues, petioles, and blades showed a higher overlapping between samples harvested in the winter months, whereas samples collected in the warmer months are better discriminated (Figure 2c,d). Perennial plants, such as *C. cardunculus*, usually have a dormant period during the colder months, when the plant considerably increases its resource reserves to ensure growth, flowering, and final yield in the upcoming growing season [32,33]. Thus, the plant’s life cycle may be the reason for such overlapping discrimination in the samples harvested during the winter months. There is also a clear discrimination in the samples of heads, seeds, and bracts harvested in the summer and spring months. This discrimination is probably justified by the weather conditions associated with each of these periods. Between the months of April and May, a dry period in the analyzed year, temperatures increased considerably from 14 to approximately 22 °C. In turn, between June and August, the temperature did not fluctuate so drastically, ranging from 21 to 26 °C [20]. Furthermore, in terms of discriminating variables, the heads, seeds, and petioles are mostly discriminated by individual fatty acids, which also account for most of the variation, while the blades and bracts seem to be characterized by the variation in polyphenols. This could be related to the function of each tissue in the plant, depending on the presence of reserve cells, structural cells, and cells used for specific biochemical functions. The obtained conclusions are in agreement with the studies described in the literature, namely Pandino et al. [34], who emphasized the great influence of weather conditions on plant phenology and quantitative and qualitative compositional characteristics. Also, Wahba et al. [35] described different levels of bioactive compounds in cardoon leaves harvested at different stages of development, with samples harvested at more advanced stages exhibiting the highest content of carbohydrates and polyphenols.

The samples analyzed in this study belong to the same plant crop and were harvested in central Greece, which allows a direct comparison between the different plant tissues and development stages studied. However, given the importance of environmental conditions, further studies with plants from different harvests would be interesting to better understand how the chemical composition and the bioactive properties of the species are influenced.

### 3.2. Principal Components Analysis

A principal components analysis (PCA) was applied to each of the plant tissues in order to reduce the dimensionality of the data and identify the most important parameters, which could be determinants in characterizing the tissues. This analysis allows samples to be clustered and, consequently, grouped between types of results (e.g., individual chemical composition and biological properties).

**Figure 3:**
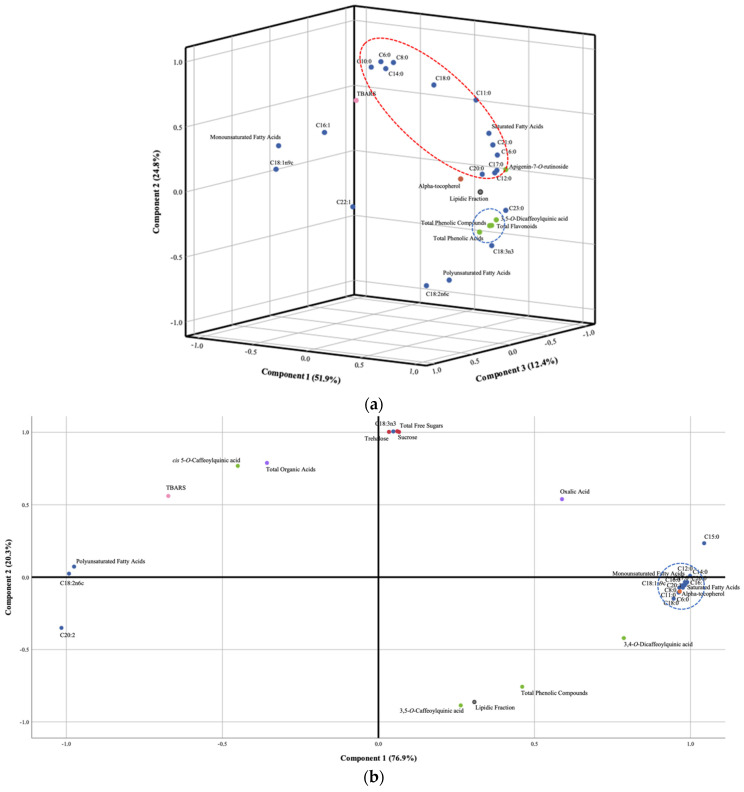

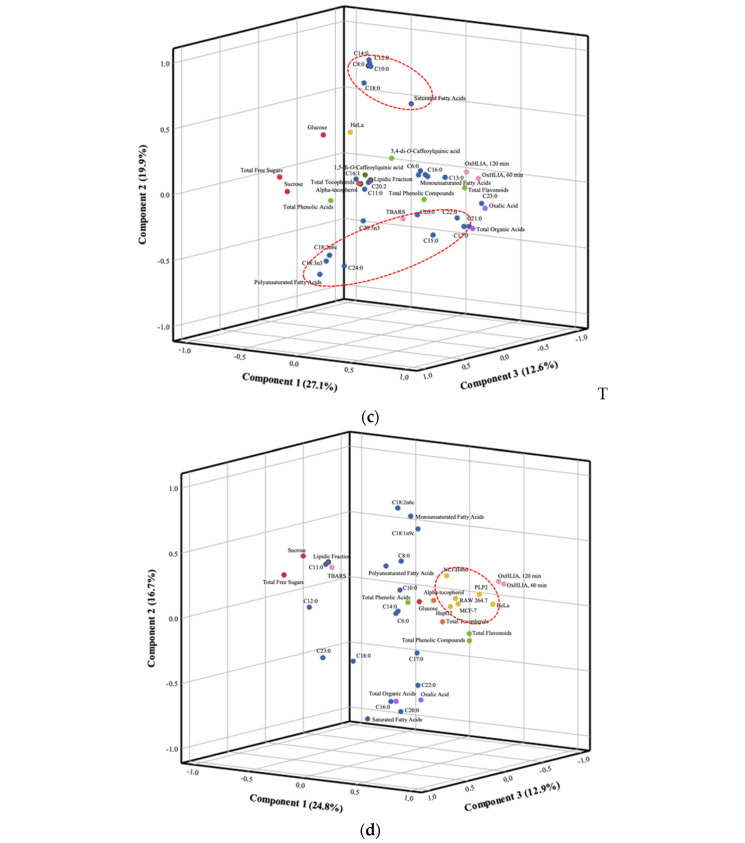

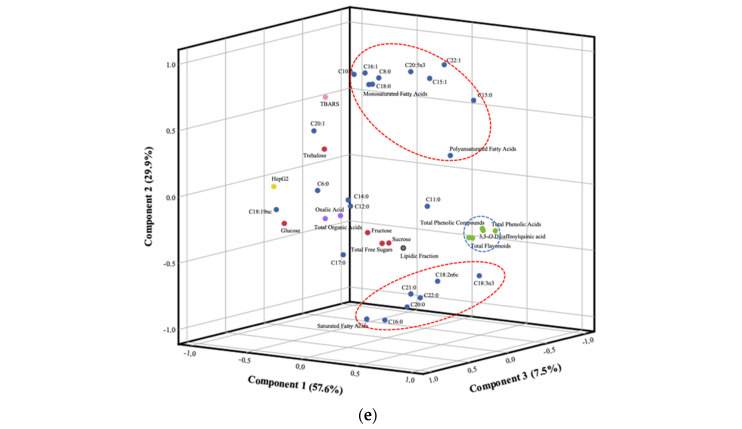
Canonical discriminant functions coefficients defined from the evaluated parameters plotted to highlight the effects of harvest time on *Cynara cardunculus* L. vegetable tissues: heads (**a**), seeds (**b**), petioles (**c**), blades (**d**), and bracts (**e**). Blue dots—fatty acids, MUFA, and PUFA; green dots—phenolic compounds; orange dots—tocopherols; red dots—soluble sugars; purple dots—organic acids; pink dots—antioxidant activity assays; yellow dots—antitumor activity.

In the case of heads’ samples, using the Kaiser–Meyer–Olkin measure of sample adequacy (KMOMSA) allows us to understand the adequacy of a PCA for the data. A result of 0.879 was obtained, meaning that it is adjusted (values closer to 1 are the most adequate); furthermore, Bartlett’s test of sphericity (BTS; another test of the adequacy of the data to a PCA analysis) was also significant. In terms of components, three components were chosen based on the eigenvalues of a scree plot (first: eigenvalue = 14.5, explained variance = 51.9%; second: eigenvalue = 6.9, explained variance = 24.8%; third: eigenvalue = 3.5, explained variance = 12.4%). Overall, the three components explained a cumulative total of 89% of the variance. Figure 3 shows the plots of all analyzed tissues, where the different parameters are colored based on the type of analysis. Blue dots represent fatty acids, MUFA, and PUFA; green dots represent individual and grouped phenolic compounds; orange dots represent individual tocopherols; red dots represent soluble sugars; purple dots represent organic acids; pink dots represent antioxidant activity assays; and yellow dots represent different cell lines used for the evaluation of the antitumor activity.

Specifically, Figure 3a shows the plots of the heads and clear discrimination of the fatty acids, which are divided into two main groups: the acids C16:1, C18:1n9c, C22:1, and MUFA, which are discriminated mainly by component 3, whereas the acids C6:0, C8:0, C10:0, C11:0, C14:0, C16:0, C18:0, C21:0, and SFA are mainly discriminated by component 2 and clustered by their composition, namely their degree of saturation. Phenolic compounds (green) were also discriminated by component 2, although they were grouped together.

For cardoon seeds, the applied function defined two dimensions (first: eigenvalue = 24.6, explained variance = 76.9%; second: eigenvalue = 6.5, explained variance = 20.3%) (Figure 3b). The KMOMSA was set at 0.526, and the BTS was significant. Component 1, responsible for 76.9% of the variability, caused an evident separation of the lipophilic components identified in the seeds (i.e., fatty acids and tocopherols), with the PUFA and two individual unsaturated fatty acids being clustered on the negative quadrant of the plot and all other lipophilic parameters being clustered on the extreme positive quadrant. Phenolic compounds and free sugars were also discriminated by component 2, exhibiting lower discriminating power than the other compounds identified in cardoon seeds. Once again, the individual fatty acids were the parameters that better clustered the plots, as in the case of heads.

Five dimensions were defined for the petioles’ samples (first: eigenvalue = 11.4, explained variance = 27%; second: eigenvalue = 8.3, explained variance = 19.9%; third: eigenvalue = 5.3, explained variance = 12.6%; fourth: eigenvalue = 4.0, explained variance = 9.6%; fifth: eigenvalue = 3.2, explained variance = 7.6%) (Figure 3c). The KMOMSA for petioles was 0.792, and the BTS was also significant. The first three principal components explained approximately 60% of the total variability. The results from the PCA analysis suggest that most of the variables are distributed over the axis of component 1, located in the most central part of the plot. In turn, the fatty acids C8:0, C10:0, C12:0, C14:0, C18:0, and SFA are grouped in the upper part (top red encirclement), mostly separated by component 2. Fatty acids C15:0, C17:0, C18:2n6c, C18:3n3, C20:0, C20:5n3, C21:0, C22:0, and C24:0 are also discriminated by component 2 in a very similar manner (bottom red encirclement), but on the negative quadrant of the plot. Moreover, some of these fatty acids are more discriminated by component 1 (e.g., C15:0, C17:0, C20:0, C21:0, and C22:0), while others are more discriminated by component 3 (e.g., C24:0, C18:2n6c, C18:3n3, and C20:5n3). The soluble sugars (red dots) were also clustered close together on the negative quadrant of component 1, while organic acids were grouped on the right quadrant of the same component (purple dots).

Regarding the blades, showing a KMOMSA of 0.745 and a significant BTS, four dimensions were defined (first: eigenvalue = 9.2, explained variance = 24.8%; second: eigenvalue = 6.2, explained variance = 16.7%; third: eigenvalue = 4.7, explained variance = 12.9%; fourth: eigenvalue = 4.2, explained variance = 11.2%). As shown in Figure 3d, the individual fatty acids detected in this tissue are scattered along component 2, while clustering has also occurred for the antitumor assays (yellow dots circled in red). The soluble sugars were also clustered on the plot, in the negative quadrant of component 1.

In the case of cardoon bracts, the PCA defined three dimensions (first: eigenvalue = 16.1, explained variance = 41.2%; second: eigenvalue = 11.5, explained variance = 29.5%; third: eigenvalue = 5.0, explained variance = 12.7%) (Figure 3e), with a KMOMSA of 0.848 and a significant BTS. The individual phenolic compounds, as well as their classes, are grouped together on the right quadrant of component 1 (blue circle), which accounts for 57.6% of the observed variability. The fatty acids are divided into two major groups, one located at the top (e.g., C8:0, C10:0, C15:0, C15:1, C18:0, C22:1, C20:5n3, C16:1, MUFA, PUFA) (top red circle) and the other at the bottom (C16:0, C20:0, C21:0, C22:0, C18:2n6c, C18:3n3, SFA) (bottom red circle) of the plot, although some other individual fatty acids are scattered along component 2.

Based on the results obtained from the PCA analysis for the various tissues, phenolic compounds have an important influence on the heads, blades, and bracts. In all these tissues, the phenolic compounds are grouped very closely and are essentially discriminated by component 1, which represents higher discriminatory power (higher percentages of discrimination). This study provides statistical evidence for the significance of the phenolic compound profile in the species as well as the influence that different stages of development may have on their profile. These conclusions agree with several studies previously published, pointing out the importance of phenolic compounds and their bioactive effects on cardoon properties [36,37]. In the case of the blades, the antiproliferative activity assays also exhibited a discriminating effect and were also discriminated by components 1 and 2. The lipophilic compounds are the ones that demonstrated the most discriminatory effect in the seeds, clustering together with the rest of the variables. Cardoon seeds are widely recognized as a rich source of vegetable oil and are being explored for human consumption and biodiesel production [15,37]. Individual fatty acids also tended to cluster in the heads, seeds, petioles, and bracts, showing their discriminant ability.

### 3.3. Cluster Analysis

**Figure 4:**
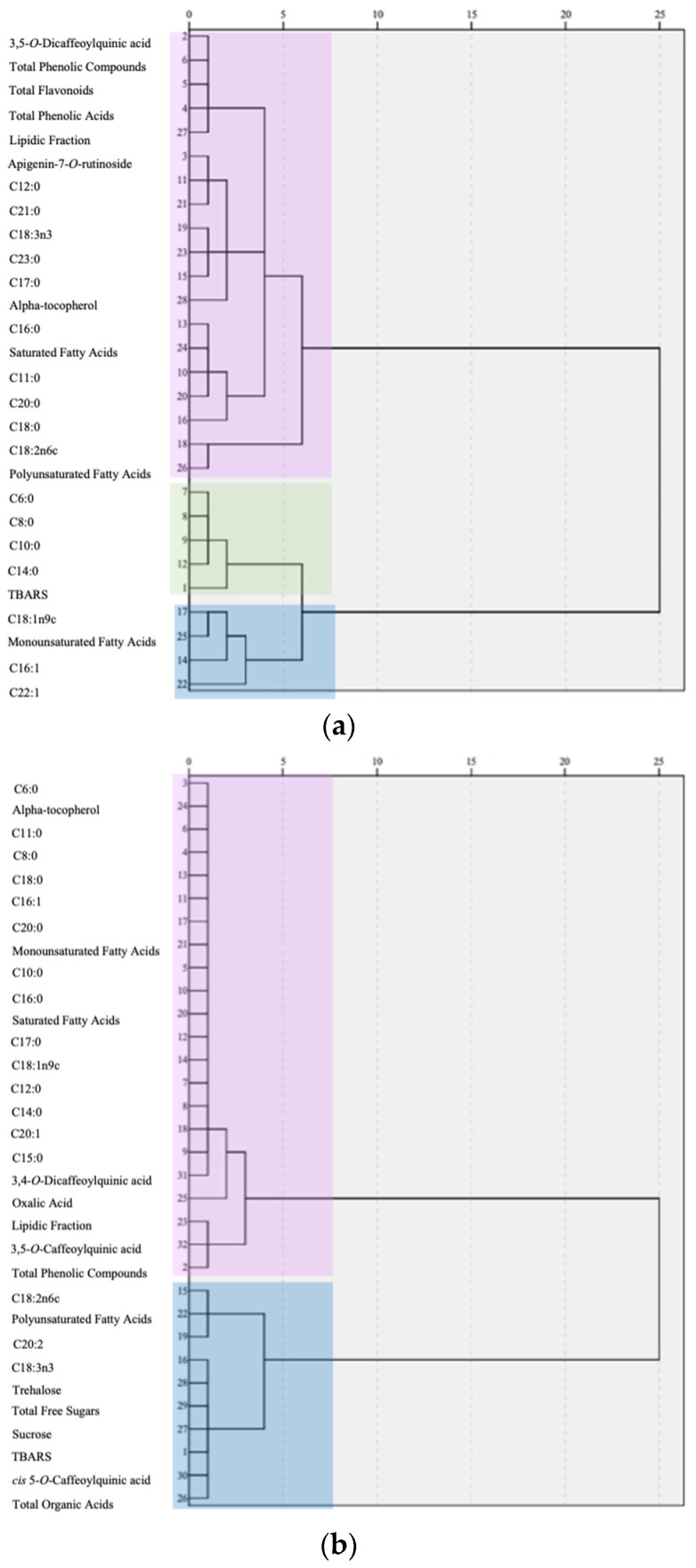

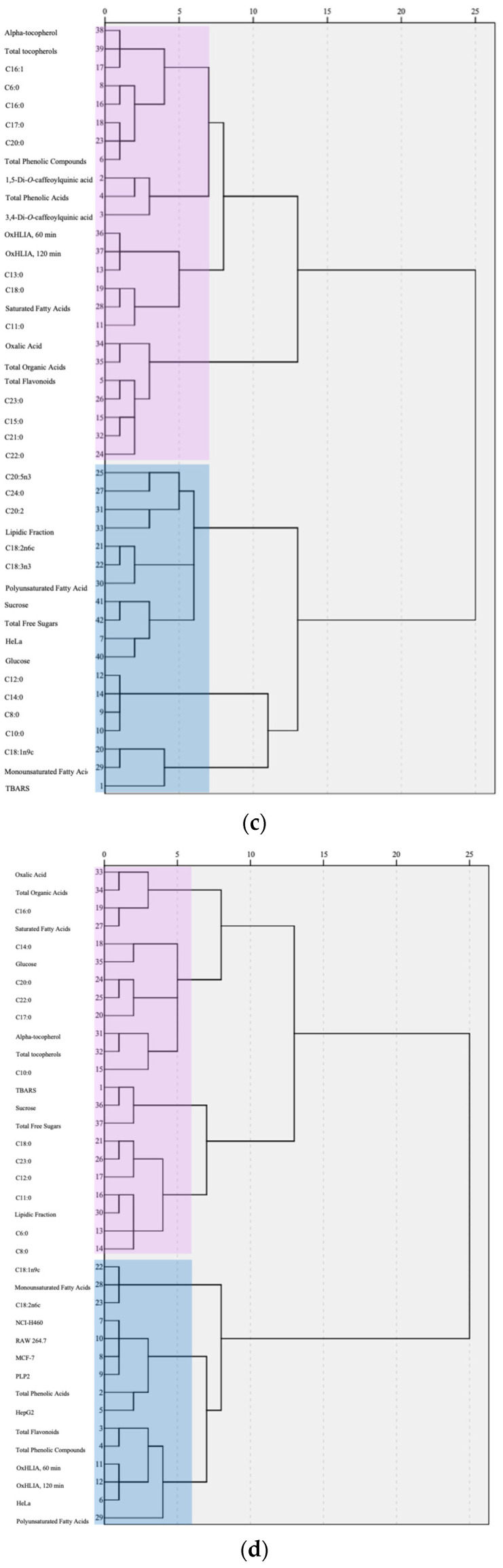

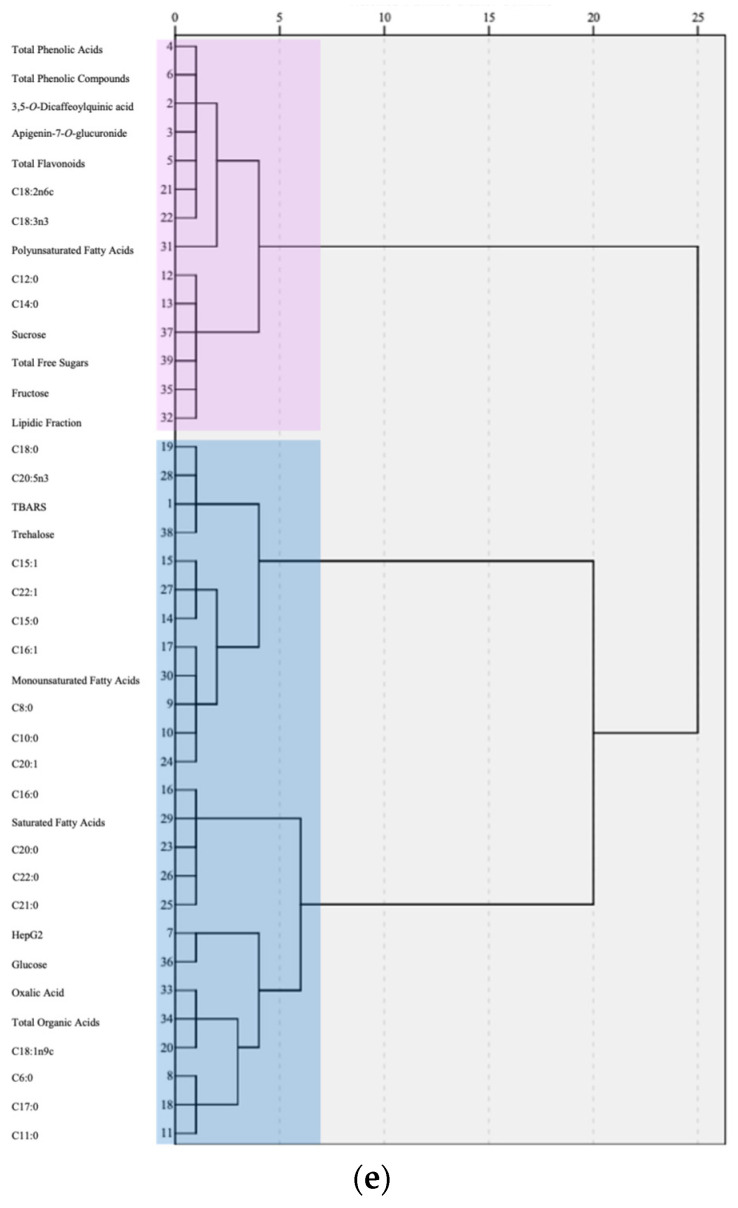
Hierarchical clustering dendrograms of *Cynara cardunculus* L. vegetable tissues: heads (**a**), seeds (**b**), petioles (**c**), blades (**d**), and bracts (**e**). Different colors in the figure represent different clusters.

Cluster analysis allows for grouping data based on their similarity, which may help identify relationships among them. There are several ways to perform this type of analysis, which may provide different cluster allocations. In the present work, the cluster analysis is intended to reveal how the different studied parameters are grouped within the distinct cardoon tissues. With this aim, a hierarchical cluster analysis (HCA), where the user defines the cluster, was performed, followed by a two-step cluster analysis (2SC), in which the clusters are defined by the algorithm of the software. Figure 4 shows the dendrograms obtained from the HCA. The representation for cardoon heads is presented in Figure 4a, where three clusters are indicated, colored pink, green, and blue. The biggest cluster (pink) includes 19 parameters, while the green and blue clusters contain 5 and 4 parameters, respectively. The blue cluster comprises only monounsaturated fatty acids, while the green one accounts for individual fatty acids and the TBARS antioxidant assay. The rest of the analyzed parameters are joined in the pink cluster. The 2SC also defined 3 clusters with “good quality”, in which the biggest cluster accounted for 50% of the total size, the second for 33.3%, and the smallest one only for 16.7%. Regarding predictor importance, the three parameters with the highest importance in terms of cluster membership were C23:0 (tricosanoic acid), monounsaturated (MUFA), and C8:0 (caprylic acid) fatty acids. Once again, the fatty acids showed high discriminant ability for the heads of cardoon.

Figure 4b represents the dendrogram for seeds, defined by two large clusters; the biggest one (highlighted in pink) is composed of 22 parameters, while the other (blue cluster) comprises only 10 parameters. In terms of the parameters, unlike the heads, both clusters include fatty acids in their composition, while all the soluble sugars are grouped into the smallest one. In the 2SC, two clusters were defined by the algorithm with a “good” classification score. They were quite different in size, with one accounting for 75% of the data and the second for only 25%. All three best predictors were liposoluble molecules, with α-tocopherol being the best predictor of cluster membership, followed by C6:0 and C8:0 fatty acids, i.e., hexanoic and caprylic acids. Like heads, fatty acids, especially caproic acid, were a good predictor of cluster membership for the seeds.

The dendrogram for the petioles of cardoon is represented in Figure 4c, where two clusters are defined, one highlighted in pink and composed of 24 parameters and the other of 18 parameters (blue). Although these clusters were quite similar in terms of size, the fatty acids were not grouped together but distributed along both clusters. Interestingly, the separation of fatty acids into different clusters, except for C16:1 (palmitoleic acid), seemed to be made according to their saturation level, with the biggest cluster including all unsaturated fatty acids. For their part, soluble sugars were clustered together in the blue cluster, whereas tocopherols (both isoforms) were included in the larger one, as well as the different polyphenols and organic acids. The 2SC showed an output of two clusters with a quality outcome of “fair” and a very different distribution of parameters when compared to the HCA. The biggest cluster included 75% of the data, and the second one only included 25%. The best predictors of cluster membership were OxHLIA 60 (erythrocyte hemolysis after 60 min), the sum of saturated fatty acids (SFA), and C6:0 (hexanoic acid). Although there was agreement in terms of cluster numbers between HCA and 2SC, the latter showed only a fair quality, and thus the results obtained for HCA are more reliable.

Figure 4d shows the clustering for cardoon blades, with two clusters through the HCA, as in the case of petioles. The clusters are also balanced, with the largest one (highlighted in pink) including 22 parameters and the other 15. Individual saturated fatty acids and their sum were included in the first cluster, while the two unsaturated fatty acids with 18 carbons and the sum of MUFA and PUFA were included in the second cluster. The 2SC for the blades showed a 3-cluster output with very similar sizes, with the quality only scoring “fair”. The biggest cluster accounted for 37.5% of the data, while the other two showed 31.3% of the data. In terms of predictors, the antioxidant activity assays (OxHLIA 60 and TBARS) showed two of the three best predictors, followed by total soluble sugars, which are all together the best parameters for cluster membership. Despite the discrepancy in clustering between the two analyses, instead of repeating the HCA considering 3 clusters, the disparity was purposely maintained since more robust results were obtained with the 2-cluster HCA distribution than with the 2SC analysis.

The final tissue subjected to cluster analysis was the cardoon bracts (Figure 4e). This was the only tissue that showed a first cluster (pink; 14 parameters) smaller than the second one (blue; 25 parameters), although there does not seem to be any coherent tendency as most groups of parameters are scattered along the two clusters. The 2SC divided the data into three clusters, classified as “good”. The smallest of them accounted for 25% of the data, while the other two had the same size, accounting for 37.5%. The best predictors for cluster membership were the saturated fatty acids, namely C16:0 (palmitic acid), C17:0 (margaric acid), and C20:0 (arachidic acid). As with the blades, a discrepancy was found between HCA and 2SC in the number of clusters, although in this case, the 2SC showed good cluster quality. Still, due to the 2SC algorithm not being known, and the defined parameters of HCA allowing for improved confidence, the discrepancy was maintained.

Overall, considering cluster analysis, discrepancies were only found for blades and bracts, while the other three cardoon tissues (heads, seeds, and petioles) showed a similar number of clusters in both HCA and 2SC analyses. Interestingly, the tissues that are not highly differentiated (bracts, blades, and petioles) were separated into two clusters, while the heads (composed of several types of tissues) and the seeds were clustered in three. This might suggest that the separation could be related to the extent of tissue differentiation in heads and seeds, while for petioles, bracts, and blades, although different from each other, the difference is less pronounced.

### 3.4. Heatmaps

**Figure 5:**
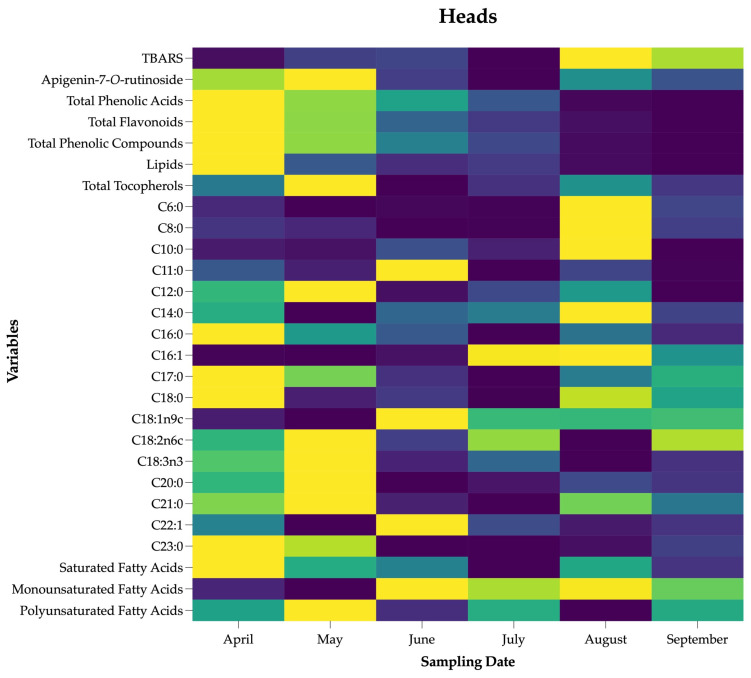

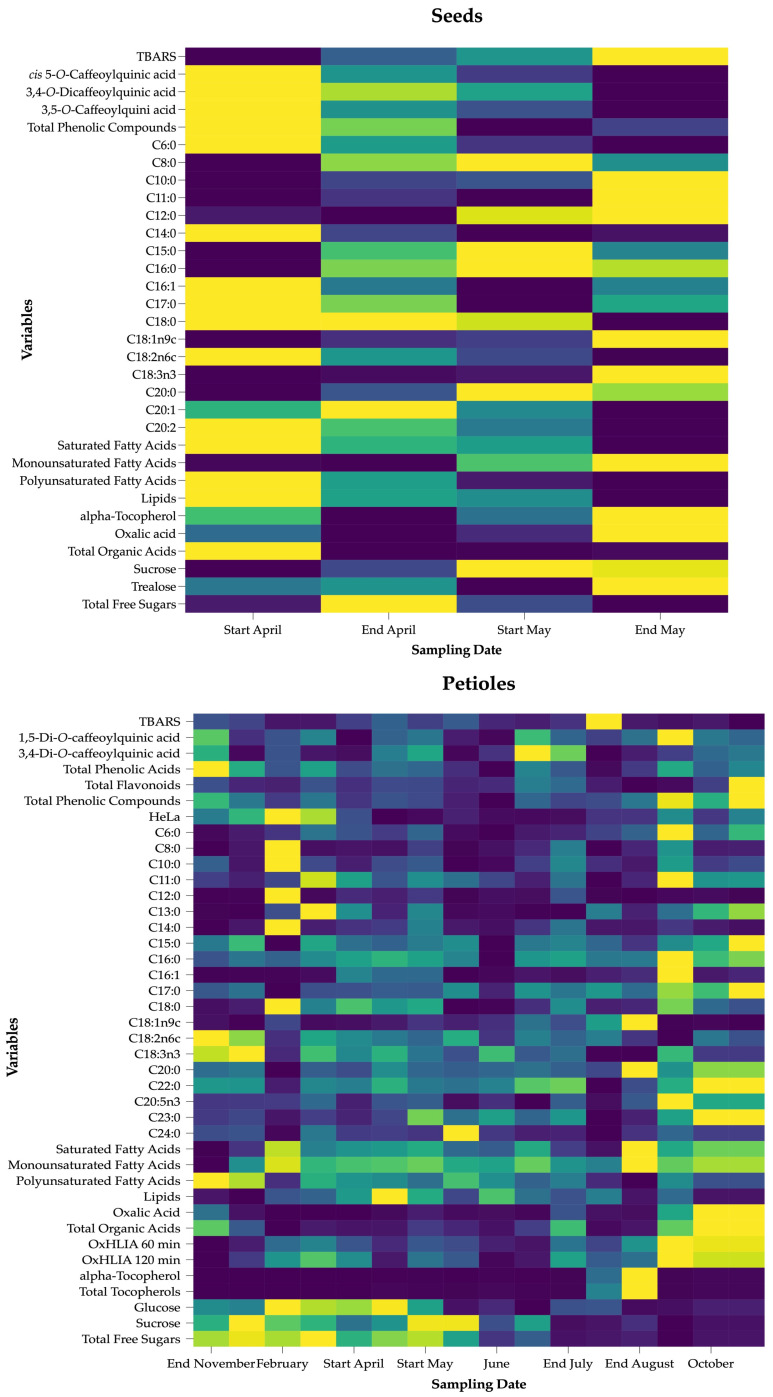

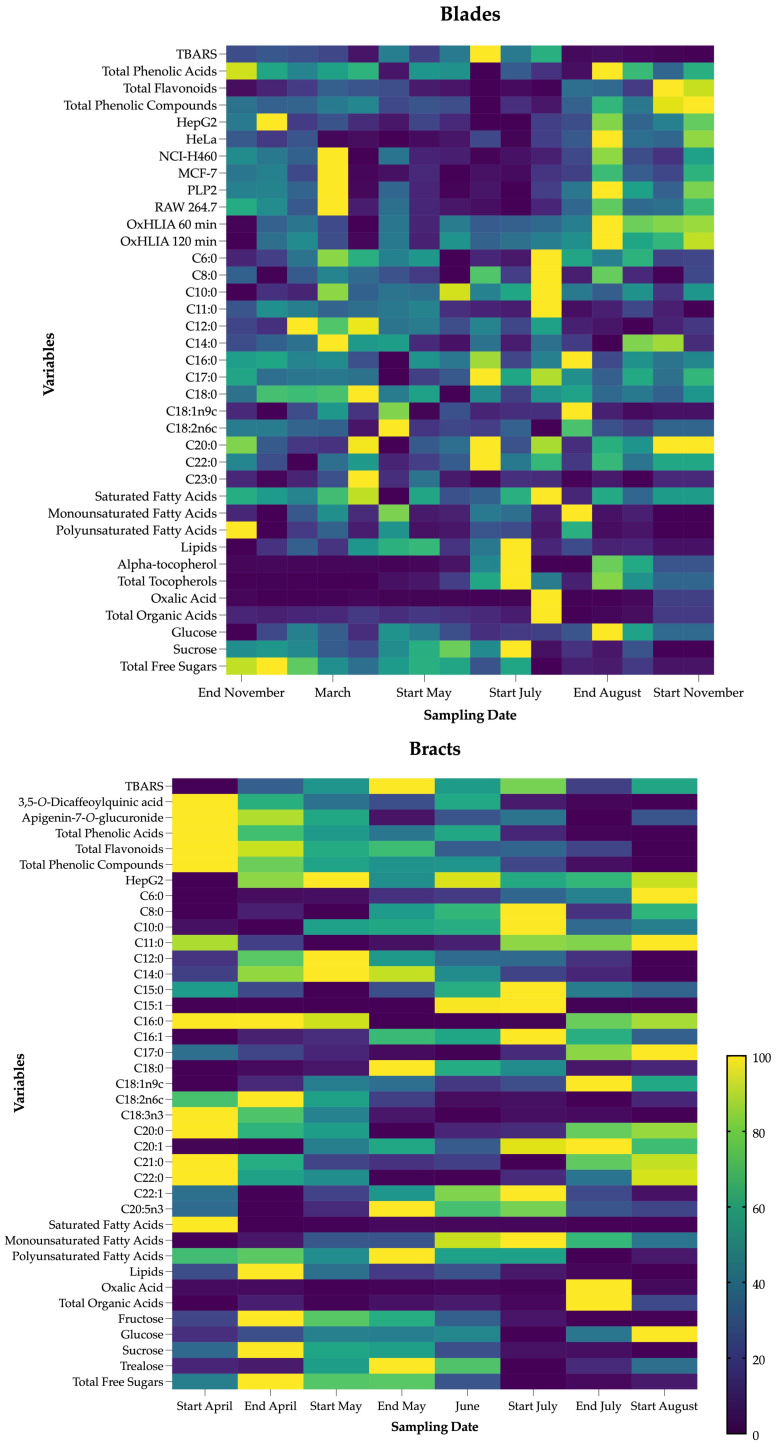
Heatmaps of the effects of harvest time on *Cynara cardunculus* L. vegetable tissues.

The heatmaps of each of the plant tissue is shown in Figure 5.

Harvesting dates are placed in columns, while the results of chemical composition and biological properties are in rows (representing the different analyzed parameters). These maps allow for an easier understanding of the seasonal fluctuation of a given parameter. The color scale characterizes the different levels of activity of each of the variables. In the case of chemical composition, darker gray scales represent higher concentrations of the individual compounds or classes, while for bioactivities, higher IC_50_ or GI_50_ values lead to lighter colors, as they stand for lower activity. For the antioxidant activity determined by the TBARS assay, the heads (Figure 5), seeds (Figure 5), and bracts (Figure 5) showed more promising activity at the beginning of April, while the petioles (Figure 5) and blades (Figure 5) stood out between the beginning of August and the beginning of November. In the case of the OxHLIA assay, petioles and blades showed the most interesting results at the end of November. Curiously, all the plant tissues studied, except the petioles, showed a higher content of phenolic compounds at harvesting dates, coinciding with the most promising antioxidant activity assessed by the TBARS assay. This observation would be in agreement with the fact that many phenolic compounds have been consistently reported as powerful antioxidant agents [9,38]. Furthermore, during these harvesting dates, temperatures start to increase, and water availability is lower, increasing the stress that plants have to endure and promoting the synthesis of secondary metabolites, such as polyphenols, which are known to be involved in the mechanisms of natural plant resistance [39].

Regarding the lipophilic compounds, lipid contents vary along the different growth stages of the studied tissues. In general, a decrease in their levels is observed in more advanced stages of growth (Figure 5). Since fatty acids are used as energy storage, the observed differences may be related to the energy requirements of plants throughout the development [19]. The tocopherol content in the heads, seeds, petioles, and blades is higher between the end of April and the end of August. Similar to polyphenols, it is well established that these compounds are important antioxidant agents that can contribute to the antioxidant defense system of plants [40,41]. A possible explanation for the observed concentration rise is their accumulation during the period when sun exposure and temperature increase drastically [40,42]. The dates with the highest tocopherol content also coincide with the highest monounsaturated fatty acid content, pointing out the protective effects of tocopherols against lipid peroxidation [23,42]. A decrease in the content of phenolic compounds between the end of April and the end of August was also observed. Given the important function that tocopherols and polyphenols play in the antioxidant system of cardoon, the observed increase in tocopherols may compensate for a decline in polyphenol content and simultaneously ensure the plant’s protection from abiotic stress [41,43].

Regarding organic acids, seeds and petioles exhibited higher contents in younger tissues, while blades and bracts reached them at the end of July (higher maturation degree). This difference may be related to the functions of each of these plant tissues. In the case of seeds and petioles, it may be related to the supply of substrate for the redox balance of cells, which can be converted into other organic acids via the tricarboxylic acid cycle. For their part, the blades and bracts are organs more involved in photosynthesis; therefore, the organic acids may be used as storage pools of fixed carbon, with their biosynthesis being associated with the incomplete oxidation of the products of photosynthesis [44,45]. In turn, the free sugar content decreases with growth progression, except for the seeds. Since the seeds are responsible for the perpetuation and multiplication of the species, this difference can be explained by the increase in energy reserves or the start of flowering of the inflorescences (associated with PSG 6), as described for other wild species under stress conditions [46]. In the other tissues studied, in addition to the fact that sugar reserves can be transferred to other plant tissues, such as seeds, more stressful environmental conditions can increase osmolyte requirements for stress tolerance mechanisms [22]. Another reason for the observed decrease may be related to the process of lignification, which results from higher sugar consumption [47]. Observing the tissues individually, in cardoon heads (Figure 5), there is a clear decrease in polyphenols over time, with a pronounced descent from May onwards. Inversely, smaller saturated fatty acids, C6:0, C8:0, and C10:0, show higher values at the end of July, while longer SFA like C20:0, C21:0, and C23:0 are more predominant in April and tend to decrease over time. In terms of polyphenols, the same behavior recorded for the heads was found in the seeds (Figure 5), showing high levels at the beginning of April and very low quantities at the end of May.

The heatmaps for petioles, blades, and bracts (Figure 5) represent a longer time period (i.e., the whole growth cycle), which makes them harder to interpret. However, some interesting features seem to be missing. At the end of the analysis, in October, the petioles showed the highest quantity of organic acids and lower antioxidant activity (OxHLIA 60 and 120), while during the other months, the values were far lower and much more constant. On the other hand, starting from November, soluble sugars start a slow and constant decrease, probably due to the slowing down of plant growth and colder temperatures. The blades, in turn, show a complete absence of tocopherols from November to May, when they start increasing, and a similar trend was observed for oxalic acid. Finally, the bracts and the seeds show a high concentration of polyphenols (focusing on the most abundant ones determined in this work, as there are several others present in lower quantity), which tend to reduce over time, especially from May onwards. Interestingly, organic acids are only present in high amounts during July, coinciding with the end of the flowering period, after which they are significantly reduced immediately. In summary, the heatmaps are interesting to quickly detect compositional patterns in the different tissues of cardoon, as well as to show how the different plant stages correlate with biotic and abiotic factors and influence the different analyzed parameters.

### 3.5. Chemical Composition and Bioactive Properties Correlation

The correlation coefficients (*R*) obtained through a Pearson correlation analysis for each studied vegetable tissue are presented in Supplementary Materials, Tables S1–S5. Moderate to strong correlations were obtained. Different shades of gray were used to highlight each of them: darker gray marked very strong correlations (*R* values higher than 0.9), gray strong correlations (*R* values between 0.7 and 0.9), and light gray moderate correlations (*R* values between 0.5 and 0.7).

For cardoon heads (Table S1), 3,5-*o*-dicaffeoylquinic acid, as well as the content of total phenolic compounds, flavonoids, and phenolic acids, demonstrate a strong negative correlation with the TBARS assay (*R* between −0.659 and 0.767), as do lipids and C18:3n3 fatty acid, which exhibit a moderate correlation (*R* between −0.584 and −0.599, respectively). Similarly, for the bracts, the phenolic acid 3,5-*o*-dicaffeoylquinic acid exhibits a negative correlation with both the TBARS assay and the HepG2 tumor cell line (−0.533 and −0.439, respectively); however, it is some of the fatty acids detected that show the strongest correlations with the TBARS assay, namely C16:0, C20:0, and C21:0 (Table S5). Also, in the blades, the most significant correlation values were observed for phenolic compounds, exhibiting negative values only with the TBARS assay (*R* between −0.559 and −0.741) (Table S4). Seeds are the plant tissue studied with the most significantly correlated *R* values for practically all the compounds detected (Table S2). In general, the phenolic acids *cis* 5-*o*-caffeoylquinic acid, 3,4-*o*-dicaffeoylquinic acid, and 3,5-*o*-caffeoylquinic acid, the total phenolic compound content, as well as the lipids and the C18:0 fatty acid, showed very strong correlation values (*R* between −0.903 and −0.988). In contrast, the sugars and total organic acids showed strong positive correlation values (*R* between 0.722 and 0.875), which suggests that they do not positively contribute to the seeds’ antioxidant capacity assessed by the TBARS test.

In general, phenolic compounds exhibit a negative correlation with the bioactive properties studied, except for blades, for which negative values were obtained only for the TBARS assay. Negative correlation values are associated with bioactivities expressed in IC_50_, and therefore, higher concentrations of phenolic compounds are associated with more potent biological activities. The results obtained suggest that the presence of phenolic compounds is an important contributor to the ability to neutralize free radicals, which is in line with previous studies that praise the crucial role of hydroxyl and carboxyl radicals in antioxidant activity [9,38].

## 4. Conclusions

Despite the large number of studies that have been carried out aiming to better understand the influence of different stages of maturation on the chemical composition and bioactive properties of cardoon, analyses and comparisons of results using in-depth statistical tools are still scarce. The results obtained in this study may contribute to clarifying how different compositions and bioactive parameters are affected over the growing period. The influence of the different seasons on the metabolism and, consequently, on the chemical composition of plant tissues has been clearly shown. A clear separation between samples harvested in the hottest and coldest months of the year was observed. Moreover, it has been evidenced that phenolic compounds may play a significant discriminating role in practically all plant tissues, except for seeds, where lipophilic compounds (especially fatty acids) have more discriminating characteristics. Specifically, in terms of statistical methods, the LDA’s allowed for an understanding of how the different molecules present in each tissue vary over time and which months show similitude between each other. In terms of understanding how samples were separated, the PCA showed the best visual cue by clustering the ones that most discriminate in each tissue. This is further supported by the cluster analysis, while the heatmaps allow us to specifically observe how each variable behaves over time in all tissues. Taking into consideration the variability of the annual variation in abiotic factors, this work could be improved with more months of analysis. Still, overall, it shows how seasonal variation occurs in most tissues of cardoon.

## Figures and Tables

**Table 1:** Harvest data and ripening stages of the studied *C. cardunculus* L. vegetable tissues.

Sampling Date	Heads	Bracts	Seeds	Petioles	Blades	PGS
September				P1	B1	1
October				P2	B2	1
Start November				P3	B3	1
End November				P4	B4	2
January				P5	B5	3
February				P6	B6	3/4
March				P7	B7	4
Start April	Car A	C1		P8	B8	4/5
End April	Car B	C2		P9	B9	5
Start May	Car C	C3		P10	B10	5/6
End May		C4	S1	P11	B11	6
June	Car D	C5	S2	P12	B12	6/7
Start July		C6	S3	P13	B13	7/8
End July	Car E	C7	S4	P14	B14	8
Start August	Car F	C8		P15	B15	8/9
End August				P16	B16	9

PGS—principal growth stages. PGS of the samples according to the Biologische Bundesanstalt, Bundessortenamt und Chemische Industrie (BBCH) scale.

## Data Availability

The original contributions presented in the study are included in the article/Supplementary Materials. Further inquiries can be directed to the corresponding author.

## Supplementary Materials

The following supporting information can be downloaded at: https://www.mdpi.com/article/10.3390/foods13162536/s1, Table S1: Person’s correlation coefficients (*R*) of chemical composition and bioactive properties of cardoon heads.; Table S2: Person’s correlation coefficients (*R*) of chemical composition and bioactive properties of cardoon seeds.; Table S3: Person’s correlation coefficients (*R*) of chemical composition and bioactive properties of cardoon petioles.; Table S4: Person’s correlation coefficients (*R*) of chemical composition and bioactive properties of cardoon blades.; Table S5: person’s correlation coefficients (*R*) of chemical composition and bioactive properties of cardoon bracts. Figure.
