# Supplementary material for: Effect of Different Seasons and Development Stages on the Chemical Composition and Bioactive Potential of Cardoon
Source: Foods. 2024 Aug 14;13(16):2536. doi: 10.3390/foods13162536 (PMC11354160; doi:10.3390/foods13162536)
Supplement: Supplementary file 1 [file foods-13-02536-s001.zip › foods-3089809-supplementary.pdf]

Article

Effect of Different Seasons and Development Stages on the Chemical Composition and Bioactive Potential of Cardoon

Filipa Mandim [1,2,3], Márcio Carocho [1,2,*], Spyridon A. Petropoulos [4], Celestino Santos-Buelga [3] and Lillian Barros [1,2]

[1] Centro de Investigação de Montanha (CIMO), Instituto Politécnico de Bragança, Campus de Santa Apolónia, 5300-253 Bragança, Portugal; filipamandim@ipb.pt (F.M.); lillian@ipb.pt (L.B.)
[2] Laboratório Associado para a Sustentabilidade e Tecnologia em Regiões de Montanha (SusTEC), Instituto Politécnico de Bragança. Campus de Santa Apolónia, 5300-253 Bragança, Portugal
[3] Grupo de Investigación em Polifenoles (GIP-USAL), Facultad de Farmacia, Universidad de Salamanca, Campus Miguel de Unamuno, 37007 Salamanca, Spain; csb@usal.es
[4] Laboratory of Vegetable Production, Department of Agriculture, Crop Production and Rural Environment, University of Thessaly, Fytokou Street, 38446 Volos, Greece; spetropoulos@uth.gr
* Correspondence: mcarocho@ipb.pt

Table S1. Pearson's correlation coefficients (*R*) of chemical composition and bioactive properties of cardoon heads.

	TBARS	
	R	Sig.
3,5-*O*-Dicaffeoylquinic acid	-0.724**	<0.001
Apigenin-7-*O*-rutinoside	-0.119	0.392
Total phenolic acids	-0.767**	<0.001
Total flavonoids	-0.659**	<0.001
Total phenolic compounds	-0.712**	<0.001
C6:0	0.792**	<0.001
C8:0	0.758**	<0.001
C10:0	0.611**	<0.001
C11:0	0.046	0.741
C12:0	-0.217	0.115
C14:0	0.360**	0.008
C16:0	-0.281*	0.040
C16:1	0.455**	<0.001
C17:0	0.039	0.779
C18:0	0.463**	<0.001
C18:1n9c	0.335*	0.013
C18:2n6c	-0.381**	0.004
C18:3n3	-0.599**	<0.001
C20:0	-0.240	0.080
C21:0	0.104	0.455
C22:1	-0.382**	0.004
C23:0	-0.403**	0.003
Saturated fatty acids	-0.143	0.302
Monounsaturated fatty acids	0.444**	<0.001
Polyunsaturated fatty acids	-0.441**	<0.001
Lipids	-0.584**	<0.001
Total tocopherols	-0.005	0.974

R – Pearson correlation coefficients; *significant at $p \le 0.05$; ** significant at $p \le 0.01$; darker gray - ≥ 0.9 Very strong correlation; gray - 0.7–0.9 Strong correlation; lighter gray - 0.5–0.7 Moderate correlation.

Table S2. Pearson's correlation coefficients (*R*) of chemical composition and bioactive properties of cardoon seeds.

	TBARS	
	R	Sig.
cis 5-*O*-Caffeoylquinic acid	0.964**	<0.001
3,4-*O*-Dicaffeoylquinic acid	-0.988**	<0.001
3,5-*O*-Caffeoylquinic acid	-0.903**	<0.001
Total phenolic compounds	-0.970**	<0.001
C6:0	-0.882**	<0.001
C8:0	-0.881**	<0.001
C10:0	-0.860**	<0.001
C11:0	-0.884**	<0.001
C12:0	-0.840**	<0.001
C14:0	-0.824**	<0.001
C15:0	-0.691**	<0.001
C16:0	-0.856**	<0.001
C16:1	-0.855**	<0.001
C17:0	-0.858**	<0.001
C18:0	-0.908**	<0.001
C18:1n9c	-0.874**	<0.001
C18:2n6c	0.848**	<0.001
C18:3n3	0.745**	<0.001
C20:0	-0.857**	<0.001
C20:1	-0.850**	<0.001
C20:2	0.593**	<0.001
Saturated fatty acids	-0.871**	<0.001
Monounsaturated fatty acids	-0.868**	<0.001
Polyunsaturated fatty acids	0.871**	<0.001
Lipids	-0.904**	<0.001
Alfa-tocopherol	-0.885**	<0.001
Oxalic acid	-0.188	0.271
Total organic acid	0.875**	<0.001
Sucrose	0.722**	<0.001
Trehalose	0.735**	<0.001
Total Sugars	0.727**	<0.001

R – Pearson correlation coefficients; *significant at $p \leq 0.05$; ** significant at $p \leq 0.01$; darker gray - ≥ 0.9 Very strong correlation; gray - 0.7–0.9 Strong correlation; lighter gray - 0.5–0.7 Moderate correlation.

Table S3. Pearson's correlation coefficients (*R*) of chemical composition and bioactive properties of cardoon petioles.

	TBARS		OxHLIA, 60 min		OxHLIA, 120 min		HeLa	
	R	Sig.	*R*	Sig.	*R*	Sig.	*R*	Sig.
1,5-Di-*O*-caffeoylquinic acid	-0.260**	0.002	0.073	0.387	-0.127	0.129	0.133	0.112
3,4-Di-*O*-caffeoylquinic acid	-0.277**	<0.001	-0.178*	0.033	-0.320**	<0.001	-0.241**	0.004
Total phenolic acids	-0.295**	<0.001	-0.076	0.368	-0.164*	0.049	0.425**	<0.001
Total flavonoids	-0.258**	0.002	0.413**	<0.001	0.294**	<0.001	-0.010	0.909
Total phenolic compounds	-0.300*	<0.001	0.577**	<0.001	0.366**	<0.001	0.281**	<0.001
C6:0	-0.272**	<0.001	0.625**	<0.001	0.500**	<0.001	0.248**	0.003
C8:0	-0.299**	<0.001	0.039	0.642	0.025	0.770	0.485**	<0.001
C10:0	-0.234**	0.005	0.075	0.373	0.050	0.552	0.542**	<0.001
C11:0	-0.429**	<0.001	0.458**	<0.001	0.448**	<0.001	0.293**	<0.001
C12:0	-0.165*	0.048	-0.054	0.523	0.041	0.626	0.475**	<0.001
C13:0	-0.101	0.228	0.776**	<0.001	0.878**	<0.001	0.367**	<0.001
C14:0	-0.172*	0.039	-0.085	0.309	-0.010	0.910	0.367**	<0.001
C15:0	-0.130	0.119	0.516**	<0.001	0.414**	<0.001	0.121	0.149
C16:0	-0.201*	0.016	0.525**	<0.001	0.337**	<0.001	-0.020	0.807
C16:1	-0.108	0.198	0.130	0.119	0.008	0.924	-0.047	0.578
C17:0	-0.028	0.743	0.650**	<0.001	0.405**	<0.001	-0.138	0.098
C18:0	-0.283**	<0.001	0.167*	0.046	0.208*	0.012	0.380**	<0.001
C18:1n9c	0.256**	0.002	-0.222**	0.007	-0.175*	0.036	-0.270**	0.001
C18:2n6c	0.248**	0.003	-0.336**	<0.001	-0.247**	0.003	0.101	0.228
C18:3n3	0.165*	0.048	-0.261**	0.002	-0.221**	0.008	-0.027	0.751
C20:0	-0.151	0.071	0.495**	<0.001	0.241**	0.004	-0.001	0.992
C22:0	0.342**	<0.001	0.338**	<0.001	0.261**	0.002	-0.158	0.058
C20:5n3	-0.097	0.246	-0.121	0.148	-0.113	0.176	-0.224**	0.007
C23:0	0.099	0.239	0.510**	<0.001	0.402**	<0.001	-0.311**	<0.001
C24:0	-0.023	0.782	-0.15	0.858	-0.004	0.965	-0.164*	0.050
Saturated fatty acids	-0.298**	<0.001	0.503**	<0.001	0.414**	<0.001	0.318**	<0.001
Monounsaturated fatty acids	0.182*	0.029	-0.100	0.235	-0.098	0.240	-0.272**	<0.001
Polyunsaturated fatty acids	0.143	0.086	-0.386**	<0.001	-0.305**	<0.001	-0.098	0.243
C20:2	-0.008	0.924	-0.046	0.585	0.103	0.219	-0.077	0.356
C21:0	-0.023	0.780	0.707**	<0.001	0.661**	<0.001	0.014	0.868
Lipids	0.003	0.971	-0.311**	<0.001	-0.298**	<0.001	-0.573**	<0.001
Oxalic acid	-0.043	0.607	0.735**	<0.001	0.598**	<0.001	0.020	0.813
Total organic acids	0.264**	0.001	0.545**	<0.001	0.450**	<0.001	-0.032	0.702
Alpha-tocopherol	-0.210*	0.011	0.133	0.112	-0.031	0.710	0.102	0.226
Total tocopherol	-0.216**	0.009	0.117	0.163	-0.043	0.609	0.082	0.326
Glucose	0.016	0.849	-0.241**	0.004	-0.013	0.875	0.441**	<0.001
Sucrose	-0.095	0.256	-0.546**	<0.001	-0.414**	<0.001	0.128	0.127
Total Sugars	-0.085	0.312	-0.491**	<0.001	-0.292**	<0.001	0.376**	<0.001

R – Pearson correlation coefficients; *significant at $p \leq 0.05$; ** significant at $p \leq 0.01$; darker gray - ≥ 0.9 Very strong correlation; gray - 0.7–0.9 Strong correlation; lighter gray - 0.5–0.7 Moderate correlation.

Table S4. Pearson's correlation coefficients (*R*) of chemical composition and bioactive properties of cardoon blades.

	TBARS		OxHLIA, 60 min		OxHLIA, 120 min		HepG2		HeLa		NCI-H460		MCF-7		PLP2		RAW 246.7	
	R	Sig.	*R*	Sig.	*R*	Sig.	*R*	Sig.	*R*	Sig.	*R*	Sig.	*R*	Sig.	*R*	Sig.	*R*	Sig.
Total phenolic acids	-0.559**	<0.001	0.150	0.072	0.087	0.297	0.583**	<0.001	0.455*	<0.001	0.515**	<0.001	0.541**	<0.001	0.501**	<0.001	0.517**	<0.001
Total flavonoids	-0.579**	<0.001	0.633**	<0.001	0.583**	<0.001	0.404**	<0.001	0.522**	<0.001	0.219**	0.008	0.302**	<0.001	0.388**	<0.001	0.384**	<0.001
Total phenolic compounds	-0.741**	<0.001	0.622**	<0.001	0.553*	<0.001	0.595**	<0.001	0.648**	<0.001	0.404**	<0.001	0.488**	<0.001	0.548**	<0.001	0.551**	<0.001
C6:0	-0.150	0.073	-0.061	0.471	-0.110	0.191	-0.130	0.121	-0.088	0.294	0.170*	0.042	0.224**	0.007	0.249**	0.003	0.144	0.085
C8:0	0.497**	<0.001	-0.018	0.833	0.027	0.752	0.104	0.214	0.225**	0.007	0.153	0.067	0.155	0.064	0.135	0.106	0.045	0.590
C10:0	0.337**	<0.001	0.011	0.899	0.105	0.211	-0.337**	<0.001	-0.209*	0.012	-0.058	0.490	0.013	0.878	-0.059	0.484	-0.144	0.085
C11:0	0.303**	<0.001	-0.401**	<0.001	-0.374**	<0.001	-0.092	0.271	-0.373**	<0.001	-0.129	0.124	-0.120	0.152	-0.204*	0.014	-0.183*	0.028
C12:0	0.227**	0.006	-0.553**	<0.001	-0.493**	<0.001	-0.453**	<0.001	-0.431**	<0.001	-0.90	0.285	-0.102	0.223	-0.246**	0.003	-0.175*	0.036
C14:0	-0.109	0.192	0.011	0.894	-0.176*	0.035	-0.150	0.073	-0.291**	<0.001	0.155	0.064	0.221**	0.008	0.201*	0.015	0.255**	0.002
C16:0	0.137	0.103	-0.126	0.132	-0.113	0.176	0.028	0.736	0.031	0.716	-0.122	0.144	0.058	0.493	-0.007	0.934	-0.023	0.784
C17:0	0.460**	<0.001	-0.007	0.935	0.014	0.867	-0.104	0.214	0.158	0.058	-0.238**	0.004	-0.018	0.831	-0.1	0.233	-0.177	0.034
C18:0	-0.185*	0.026	-0.362**	<0.001	-0.372**	<0.001	0.089	0.287	-0.199	0.154	0.104	0.214	0.196*	0.019	0.118	0.161	0.144	0.085
C18:1n9c	0.028	0.742	0.276**	<0.001	0.286**	<0.001	0.063	0.455	0.295**	<0.001	0.601**	<0.001	0.346**	<0.001	0.501**	<0.001	0.446**	<0.001
C18:2n6c	0.297**	<0.001	0.220**	0.008	0.231**	0.005	0.177*	0.034	0.229**	0.006	0.428**	<0.001	0.165*	0.049	0.316**	<0.001	0.321**	<0.001
C20:0	0.040	0.637	-0.113	0.179	-0.116	0.167	-0.138	0.100	0.075	0.373	-0.390**	<0.001	-0.228**	0.006	0.265**	0.001	0.265**	0.001
C22:0	0.126	0.133	-0.033	0.696	-0.057	0.500	-0.247**	0.003	-0.047	0.575	-0.340**	<0.001	-0.170*	0.041	-0.207*	0.013	-0.261**	0.002
C23:0	-0.114	0.173	-0.531**	<0.001	-0.558**	<0.001	-0.297**	<0.001	-0.328**	<0.001	-0.250**	0.003	-0.206*	0.013	-0.293**	<0.001	-0.239**	0.004
Saturated fatty acids	0.063	0.453	-0.424**	<0.001	-0.439**	<0.001	-0.160	0.056	-0.176*	0.034	-0.228**	0.036	-0.002	0.978	-0.129	0.123	-0.122	0.146
Monounsaturated fatty acids	0.163	0.051	0.228**	0.006	0.259**	0.002	-0.048	0.566	0.205*	0.014	0.464**	<0.001	0.221**	0.008	0.347**	<0.001	0.278**	0.006
Polyunsaturated fatty acids	-0.208*	0.012	0.312**	<0.001	0.304**	<0.001	0.224**	0.007	0.038	0.649	-0.116	0.165	-0.182*	0.029	-0.136	0.104	-0.089	0.288
Lipids	0.548**	<0.001	-0.358**	<0.001	-0.312**	<0.001	-0.476**	<0.001	-0.386**	<0.001	-0.436**	<0.001	-0.482**	<0.001	-0.497	<0.001	-0.551**	<0.001
Alfa-tocopherol	0.153	0.067	0.320**	<0.001	0.231**	0.005	-0.115	0.169	0.046	0.581	-0.347**	<0.001	-0.173*	0.039	-0.142	0.090	-0.276**	<0.001
Total tocopherol	0.168*	0.044	0.340**	<0.001	0.291**	<0.001	-0.189*	0.023	0.073	0.385	-0.405**	<0.001	-0.242**	0.003	-0.171*	0.040	-0.347**	<0.001
Glucose	0.235**	0.005	0.368**	<0.001	0.224**	0.007	-0.167*	0.046	0.069	0.414	-0.161	0.054	-0.121	0.147	0.005	0.950	-0.135	0.106
Sucrose	0.846**	<0.001	-0.494**	<0.001	-0.410**	<0.001	-0.395**	<0.001	-0.504**	<0.001	-0.431**	<0.001	-0.456**	<0.001	-0.603**	<0.001	-0.530**	<0.001
Total free sugars	0.508**	<0.001	-0.664**	<0.001	-0.611**	<0.001	-0.042	0.621	-0.488**	<0.001	-0.066	0.429	-0.126	0.133	-0.310**	<0.001	-0.119	0.154
Oxalic acid	-0.275**	<0.001	0.079	0.348	0.146	0.081	-0.020	0.816	0.124	0.140	-0.045	0.592	-0.026	0.755	0.081	0.334	0.031	0.712
Total organic acids	-0.117	0.163	-0.134	0.110	-0.049	0.561	-0.133*	0.028	-0.071	0.396	-0.155	0.064	-0.154	0.066	-0.086	0.303	-0.103	0.219

R – Pearson correlation coefficients; *significant at $p \leq 0.05$; ** significant at $p \leq 0.01$; darker gray - ≥ 0.9 Very strong correlation; gray - 0.7–0.9 Strong correlation; lighter gray - 0.5–0.7 Moderate correlation.

Table S5. Pearson's correlation coefficients (*R*) of chemical composition and bioactive properties of cardoon bracts.

	TBARS		HepG2	
	R	Sig.	*R*	Sig.
3,5-*O*-Dicaffeoylquinic acid	-0.533**	<0.001	-0.439**	<0.001
Apigenin-7-*O*-glucuronide	-0.204	0.086	-0.485**	<0.001
Total phenolic acids	-0.430**	<0.001	-0.395**	<0.001
Total flavonoids	-0.310**	0.008	-0.515**	<0.001
Total phenolic compounds	-0.359*	0.002	-0.433**	<0.001
C6:0	0.178	0.136	0.303**	0.010
C8:0	0.729**	<0.001	0.178	0.134
C10:0	0.775**	<0.001	0.278*	0.018
C11:0	-0.343**	0.003	-0.403**	<0.001
C12:0	0.206	0.083	0.381**	<0.001
C14:0	0.315**	0.007	0.297*	0.011
C15:0	0.082	0.495	-0.311**	0.005
C15:1	0.343**	0.003	0.160	0.179
C16:0	-0.749**	<0.001	-0.037	0.758
C16:1	0.632**	<0.001	0.062	0.605
C17:0	-0.378**	0.001	0.017	0.888
C18:0	0.811**	<0.001	-0.023	0.847
C18:1n9c	0.001	0.994	0.311**	0.008
C18:2n6c	-0.476**	<0.001	-0.174	0.145
C18:3n3	-0.662**	<0.001	-0.490**	<0.001
C20:0	-0.824**	<0.001	-0.231	0.051
C20:1	0.425**	<0.001	0.172	0.148
C21:0	-0.750**	<0.001	-0.324**	0.005
C22:0	-0.646**	<0.001	-0.252*	0.033
C22:1	0.457**	<0.001	-0.181	0.129
C20:5n3	0.690**	<0.001	-0.266*	0.024
Saturated fatty acids	-0.571**	<0.001	-0.116	0.330
Monounsaturated fatty acids	0.372**	0.001	0.318**	0.006
Polyunsaturated fatty acids	0.327**	0.005	-0.344**	0.003
Lipids	-0.249*	0.035	0.189	0.112
Oxalic acid	-0.397**	<0.001	-0.032	0.788
Total organic acids	-0.348**	0.003	0.070	0.557
Fructose	0.077	0.521	0.246*	0.037
Glucose	0.198	0.096	0.522**	<0.001
Sucrose	-0.054	0.649	0.091	0.448
Trehalose	0.612**	<0.001	0.250*	0.034
Total free sugars	0.008	0.945	0.052	0.662

R – Pearson correlation coefficients; *significant at $p \leq 0.05$; ** significant at $p \leq 0.01$; darker gray - ≥ 0.9 Very strong correlation; gray - 0.7–0.9 Strong correlation; lighter gray - 0.5–0.7 Moderate correlation.
